# Semantic Visualization in Functional Recovery Prediction of Intravenous Thrombolysis following Acute Ischemic Stroke in Patients by Using Biostatistics: An Exploratory Study

**DOI:** 10.3390/jpm13040624

**Published:** 2023-04-01

**Authors:** Chih-Chun Hsiao, Chun-Gu Cheng, Cheng-Chueh Chen, Hung-Wen Chiu, Hui-Chen Lin, Chun-An Cheng

**Affiliations:** 1 Department of Nursing, Taoyuan Armed Forces General Hospital, Taoyuan 32549, Taiwan; 2 Department of Emergency Medicine, Taoyuan Armed Forces General Hospital, Taoyuan 32549, Taiwan; 3 Department of Emergency Medicine, Tri-Service General Hospital, National Defense Medical Center, Taipei 11490, Taiwan; 4 Department of General Surgery, China Medical University Beigang Hospital, Yunlin 65152, Taiwan; 5 Graduate Institute of Medical Informatics, College of Medical Science and Technology, Taipei Medical University, Taipei 11031, Taiwan; 6 School of Nursing, College of Nursing, Taipei Medical University, Taipei 11031, Taiwan; 7 Department of Neurology, Tri-Service General Hospital, National Defense Medical Center, Taipei 11490, Taiwan

**Keywords:** functional recovery, intravenous thrombolysis, acute ischemic stroke

## Abstract

(1) Background: Intravenous thrombolysis following acute ischemic stroke (AIS) can reduce disability and increase the survival rate. We designed a functional recovery analysis by using semantic visualization to predict the recovery probability in AIS patients receiving intravenous thrombolysis; (2) Methods: We enrolled 131 AIS patients undergoing intravenous thrombolysis from 2011 to 2015 at the Medical Center in northern Taiwan. An additional 54 AIS patients were enrolled from another community hospital. A modified Rankin Score ≤2 after 3 months of follow-up was defined as favorable recovery. We used multivariable logistic regression with forward selection to construct a nomogram; (3) Results: The model included age and the National Institutes of Health Stroke Scale (NIHSS) score as immediate pretreatment parameters. A 5.23% increase in the functional recovery probability occurred for every 1-year reduction in age, and a 13.57% increase in the functional recovery probability occurred for every NIHSS score reduction. The sensitivity, specificity, and accuracy of the model in the validation dataset were 71.79%, 86.67%, and 75.93%, respectively, and the area under the receiver operating characteristic curve (AUC) was 0.867; (4) Conclusions: Semantic visualization-based functional recovery prediction models may help physicians assess the recovery probability before patients undergo emergency intravenous thrombolysis.

## 1. Introduction

In general, approximately 80% of all cerebrovascular disease cases are ischemic strokes. The subsequent management of sequelae after acute ischemic stroke (AIS) is associated with both higher medical expenses and time-consuming physical therapy. Intravenous thrombolysis can increase the survival rate and reduce disability or the length of hospital stay in patients with AIS [1,2,3]. Since some patients may have unfavorable outcomes, patients and families are concerned with the recovery probability before electing to undergo intravenous thrombolysis treatment. A total of 8.84% of stroke patients received intravenous thrombolysis from 2006–2008, as reported in the Taiwan Stroke Registry. The intravenous thrombolysis rate in AIS patients was 0.6% from 2003–2010 in Taiwan, which was lower than the 1.15% intravenous thrombolysis rate reported in the United States between 2000 and 2006 [4,5,6]. Although the method and patient education have improved in recent years, a useful tool is still required to help with rapid treatment decision making, which will reduce the in-hospital delay as the doctor and patient communicate. Functional recovery analysis may further clarify the prognosis of intravenous thrombolysis treatment. Here, we attempted to establish semantic visualization with simple numerical estimates for AIS patients considering thrombolysis treatment.

A younger age and a lower National Institutes of Health Stroke Scale (NIHSS) score are associated with a better level of recovery [2]. The DRAGON score is a functional prediction system based on immediate pretreatment parameters that has been established to predict good (modified Rankin Scale [mRS]: 0–2) or poor recovery (mRS: 5–6) with acceptable sensitivity and specificity for AIS patients; however, patients with mRS scores of 3 and 4 with support needs were missing from the evaluation [7]. The Stroke Prognostication using Age and NIH Stroke Scale (SPAN)-100 index evaluates age and the NIHSS neurological impairment score to predict the clinical response and risk of hemorrhagic complications after thrombolysis in AIS patients, but the index has limited efficacy for recovery prediction [8]. The majority of previous studies have classified only good or poor recovery situations without quantifying the degree of recovery, and thus, physicians are not able to use recovery probability data to persuade AIS patients and relatives to consider intravenous thrombolysis during the short therapeutic window. We need to develop a semantic visualization for functional recovery probability assessment that will enable stroke treatment physicians to estimate the likelihood of functional recovery before recommending intravenous thrombolysis during the early stage of AIS in the emergency room.

Nomograms are widely used for disease prognosis and support simple numerical estimated event probability. The nomogram algorithm is built based on statistically significant factors, and physicians can use the one-page nomogram to predict disease outcomes [9,10,11,12]. The nomogram algorithm assigns a point value for each potential risk factor and sums all points of all factors to acquire a total point value with which the model predicts the probability for an individual patient. Nomograms for functional recovery and survival prediction as semantic visualization have been created and fitted with logistic regression to predict outcomes after 3 months based on the age and NIHSS score criteria within 6 h after AIS symptom onset [11] and have been validated based on intracranial hemorrhage or mortality within 3 months of intravenous thrombolysis [13]. In contrast, our study used parameters in the early stage (within 3 h) to predict the outcome before patients underwent thrombolysis.

Although endovascular treatment for large vessel occlusion has been increasingly performed in recent years [14], the technology and instruments are not always available in community hospitals. It is important to predict the prognosis of treatment for personalized medicine. We developed the model with a dataset from a medical center and externally validated the model using a dataset from another community hospital. We predicted the functional recovery probability within 3 months after intravenous thrombolysis treatment of AIS in a Chinese group. We used semantic visualization for the prognostic prediction of intravenous thrombolysis treatment at individual patient care. Older and more severe AIS patients could be referred to the medical center for aggressive assessment and treatment such as thrombus removal for recanalization in large vessel occlusion.

## 2. Materials and Methods

**Figure 1:**
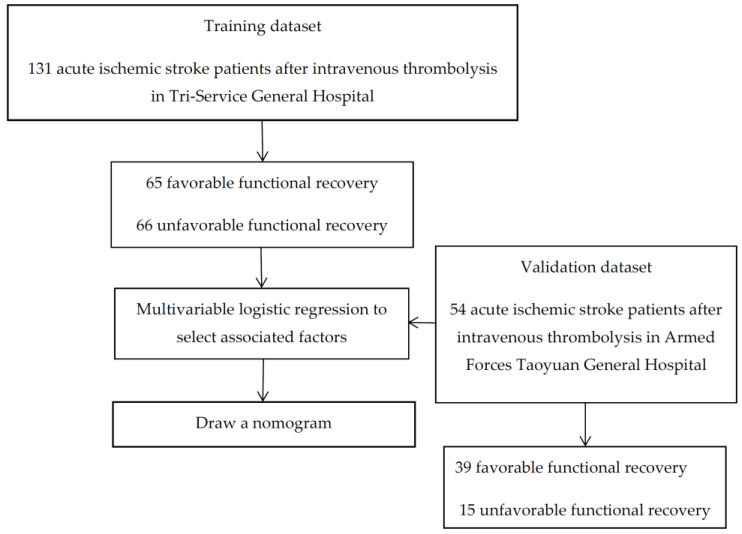
Flow chart of intravenous thrombolysis prognosis.

In this study, we collected retrospective data from 131 AIS patients who received intravenous thrombolysis treatment without contraindications within 3 h of stroke symptom onset from 1 January 2011 to 31 December 2015, in the Tri-Service General Hospital according to the Taiwan Stroke Association guidelines (no hemorrhagic stroke by brain computer tomography, onset of AIS was less than 3 h and excluded milder (NIHSS score < 4) contraindications). The validation dataset was collected by an emergency physician (C-G Cheng) from patients enrolled during the same period in Taoyuan Armed Forces General Hospital. The flowchart of this study is shown in Figure 1. The Tri-Service General Hospital is a tertiary medical center, and Taoyuan Armed Forces General Hospital is a community hospital in northern Taiwan. The study was approved by the Institutional Review Board of the Tri-Service General Hospital (Ethical Application Ref: TSGHIRB-2-106-05-116).

The variables that were collected included age, NIHSS score measured in the emergency room within 3 h, sex, onset of treatment time (OTT), estimated glomerular filtration rate (estimated GFR), internal carotid artery occlusion, hypertension (blood pressure more than 140/90 mmHg or previously received antihypertensive therapy), diabetes mellitus (fasting blood sugar more than 126 mg/dL at two readings or previously received antihyperglycemic agents), previous stroke, coronary artery disease, atrial fibrillation, anemia (hemoglobin <13 mg/dL in males and <12 mg/dL in females), and dense middle cerebral arterial sign. Intracranial hemorrhage and stroke type (large vessel, embolic, or lacunar according to magnetic resonance imaging or intracranial and extracranial sonography) were noted after hospitalization. We adopted the mRS to evaluate the stroke recovery situation; an mRS ≤ 2 without significant disability in outpatients within 3 months after intravenous thrombolysis treatment was defined as favorable functional recovery.

The data are presented as the mean ± standard deviation (SD) or as a percentage. Continuous variables were evaluated with Student’s t test, categorical variables were evaluated with the chi-square (χ^2^) test, and the NIHSS scores were evaluated with the Mann–Whitney U test because this variable is not normally distributed for comparisons between two groups. A *p*-value less than 0.05 was considered significant. The predictors of functional recovery were assessed by multivariable logistic regression with forward stepwise selection with *p* < 0.05. The effect size is 0.3883 with NIHSS. The effect size was 0.4983 with NIHSS and ‘Age’. When we added diabetes mellitus with NIHSS and age, the effect size was 0.5067; however, the *p*-value of diabetes mellitus is 0.266, so it needed to be removed. When we added NIHSS, age, diabetes mellitus, and middle cerebral artery sign, the effect size was 0.5339; however, the *p*-value of diabetes mellitus is 0.244, and the *p*-value of middle cerebral artery sign is 0.105, so both diabetes mellitus and middle cerebral artery sign needed to be removed.

The nomogram was constructed with predicted values fitted from the logistic regression model using R software version 4.0.5 with the rms package [15]. To use the nomogram as semantic visualization, each predictor was scored (range 0–100); then, the point scores of the individual variables were merged into a total point value. A line was drawn straight downward from the total point axis to the lowest line, which corresponded to an estimated recovery probability. 

We calculated the probability of the model and set the threshold to achieve sensitivity and specificity. A receiver operating characteristic (ROC) curve was constructed based on the sensitivity and false positive rate (1 − specificity). The area under the ROC curve (AUC-ROC) was used to evaluate the performance of the model, with an AUC-ROC of 0.7 considered good, 0.8 considered better, and 0.9 considered perfect performance. All statistical analyses were performed using R software version 4.0.5. An internal bootstrap validation for 200 resamples was performed to correct the overfitting bias of testing on the same patient population and to discriminate the functional recovery of two patients (overfitting-corrected concordance index [c-index] for discrimination) [16,17].

## 3. Results

The training dataset consisted of 131 AIS patients with 78 males and 53 females; the mean age was 63.64 ± 12.1 years, the median NIHSS score was 15 with a range of 7–27, and 65 patients (49.62%) had a favorable recovery. The patients with a good recovery status were younger and had lower NIHSS scores, earlier onset treatment times, lower estimated glomerular filtration rates, a more lacunar or embolic infarction type, and less atrial fibrillation. The demographic characteristics of the training dataset are shown in Table 1.

We used data from another community hospital to validate our development model. The validation dataset included 54 AIS patients consisting of 26 males and 28 females; the mean age was 63.74 ± 10.12 years, the median NIHSS score was 12 with a range of 4–26, and 39 patients (72%) had a favorable recovery. No significant differences were found in age, sex, or the survival state between the training and validation datasets, but the median NIHSS score was lower and the functional recovery proportion was higher in the validation dataset (Table 2). 

**Figure 2:**
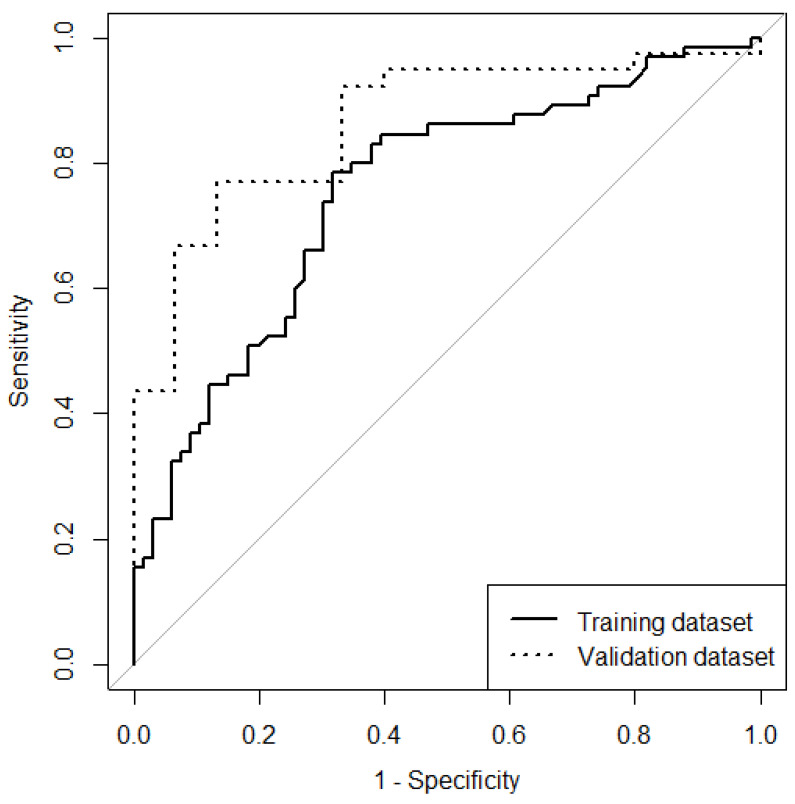
Receiver operating characteristic curve of the training and validation datasets.

An older age, greater overall neurological impairment based on the NIHSS score, less lacunar ischemic stroke type, diabetes mellitus, and a dense middle cerebral arterial sign were associated with unfavorable functional recovery in the univariate analysis. In the multivariable logistic regression analysis, the independent predictive factors of functional recovery within 3 months after intravenous thrombolysis were age and NIHSS score. The recovery probability was increased by 5.23% for every 1-year reduction in age and 13.57% for every reduction in the NIHSS score (Table 3). The sensitivity, specificity, and accuracy of the model in the training dataset were 72.31%, 69.70%, and 70.99%, respectively; the AUC-ROC was 0.753, and the corrected c-index after bootstrap validation was 0.744. The sensitivity, specificity, and accuracy of the model in the validation dataset were 71.79%, 86.67%, and 75.93%, respectively, and the AUC-ROC was 0.867 (95% confidence interval [C.I.]: 0.7653 to 0.968) (Figure 2).

**Figure 3:**
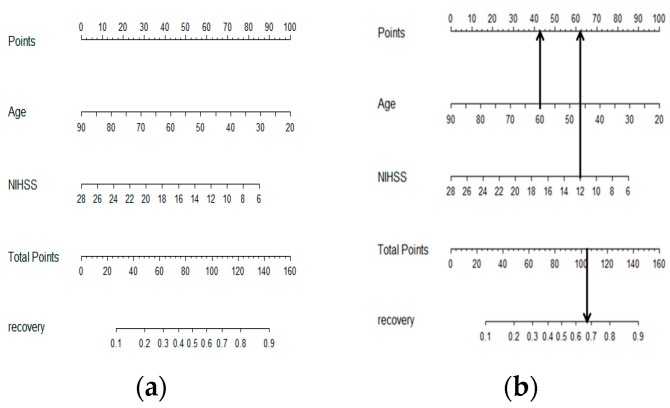
(**a**) Nomogram for functional recovery probability after intravenous thrombolysis. (**b**) Nomogram application of recovery probability before thrombolysis. A line was drawn straight upward with an up arrow to obtain the points for the variables. For example, a patient aged 60 years (43 points) had an NIHSS score of 12 (62 points). The total point score was the sum of age and NIHSS scores with 105. Drawing a straight line downward from total points 105 to the lowest line then corresponds to an estimated recovery probability of 67%.

The nomogram for semantic visualization was developed based on a multivariable logistic regression model. Age and the NIHSS score were the linear predictors used to calculate the functional recovery probability within 3 months after AIS. This prediction was determined by comparing the ‘age’ axis to the ‘points’ scale and by drawing a vertical line upward between the two axes; the same process was repeated for the ‘NIHSS’ factor. Summing the ‘Age’ and ‘NIHSS’ points gives rise to the ‘Total Points’ scale, and a vertical line drawn to the ‘functional recovery probability’ axis indicates the estimated functional recovery probability (Figure 3A). For example, a patient aged 60 years (43 points) with an overall NIHSS score of 12 (62 points) receives a total point score of 105. This score corresponded to an estimated probability of 67% for functional recovery (Figure 3B). Total points in this nomogram greater than 85 indicated a 50% likelihood of favorable functional recovery. We judged the performance and calibration of our nomogram with calibration and demonstrated similarity between the predictions of functional recovery probability after 3 months of treatment from the developmental model with the actual observed functional recovery extent in the patients in the training dataset. We determined that the nomogram was well calibrated, with a mean absolute error = 0.024 and a slope near 1.

**Figure 4:**
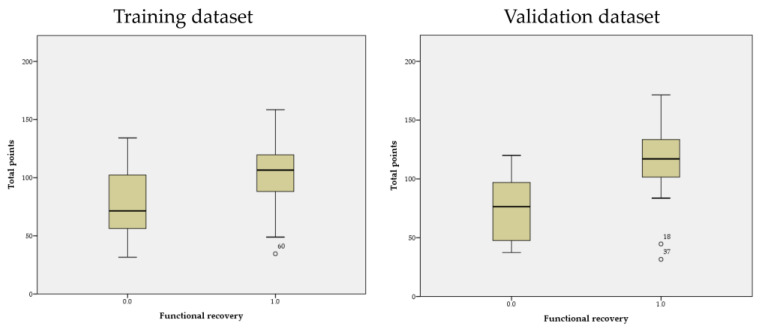
The box plots of score distribution in both event and no-event groups in the training and validation datasets.

This section may be divided by subheadings. It should provide a concise and precise description of the experimental results and their interpretation, as well as the experimental conclusions that can be drawn. The score distribution in both event and no-event groups in the training and validation cohorts is shown in Figure 4.

## 4. Discussion

The decision to undergo intravenous thrombolysis therapy requires rapid making, which causes anxiety and worry for patients and relatives regarding the outcome of the therapy. In this study, we used age and the NIHSS score before intravenous thrombolysis to develop a prediction nomogram as semantic visualization based on a medical center hospital dataset; then, the nomogram was externally validated using a community hospital dataset with good performance.

A study in England found increased intracranial hemorrhage and mortality in patients admitted to the emergency department by ambulance; indeed, the urgent care of AIS patients is a challenging task [18]. Female sex, normal blood glucose, and no cortical involvement are associated with major neurological improvements [19]. The hemorrhage after thrombolysis (HAT) score showed that patients who were older than 80 years and had a stroke area encompassing more than 1/3 of the middle cerebral artery territory were more likely to experience brain hemorrhage after thrombolysis [20]. When AIS occurs in patients older than 80 years with a stroke area encompassing more than 1/3 of the middle cerebral artery territory, intravenous thrombolysis is not recommended based on the guidelines of the Taiwan Stroke Association [21]. Patients who are recommended to receive intravenous thrombolysis according to this guideline seem to have a lower risk of brain hemorrhage. However, a previous study revealed a twofold increase in the brain hemorrhage risk after intravenous thrombolysis in an Asian cohort [22]. Furthermore, in the TTT-AIS study, more hemorrhage occurrences were reported in thrombolysis patients older than 70 years (odds ratio: 4.12) [23]. Our study found that age was related to recovery from intravenous thrombolysis. If patients suffering from a decline in the Glasgow Coma Scale score undergo brain imaging to check for intracranial hemorrhage, the time may lag several days. The patients with AIS suffering from any type of intracranial hemorrhage after intravenous thrombolysis had more unfavorable functional recovery in the training dataset (39.4% vs. 4.6%, *p* < 0.001), which was classified as poor recovery.

Using the DRAGON score with 6 categorical variables (age [younger than 65 years: 0; 65–79 years old: 1; and ≥80 years old: 2], blood sugar above 144 mg/dL, OTT > 90 min, dense cerebral arterial sign or early infarct on CT, previous stroke, and NIHSS [1 = NIHSS: 5–9; 2 = NIHSS: 10–15; and 3 = NIHSS: > 15]), a score of 0 predicts a good outcome (mRS: 0–2), and a score of 10 predicts a poor outcome (mRS: 5–6); therefore, in this scoring system, a lower score indicates a better prognosis (7). The iScore predicts the effectiveness of thrombolysis for AIS patients using a complex calculation method [24]. Age (A), severity of stroke (S) measured by the admission NIH Stroke Scale score, stroke onset to admission time (T), range of visual fields (R), acute glucose level (A), and level of consciousness (L) are considered in the ASTRAL (Acute Stroke Registry and Analysis of Lausanne) score, which exhibits good performance [25]. One study compared the scoring systems with the physicians’ judgment and found that the ASTRAL and DRAGON scores were more accurate for stroke outcome prediction [26]. The Alberta Stroke Program Early CT Score (ASPECTS) evaluated brain computed tomography changes at the early stage of middle cerebral artery ischemic stroke and showed that earlier changes assessed by computed tomography were associated with a poorer outcome [27]. The TURN score found that a lower NIHSS score, higher prestroke activity condition, and higher platelet count were associated with a lower intracranial hemorrhage risk and good recovery [28]. The SPAN-100 index combined age and the NIHSS score for simple clinical use; however, this index had a limited performance ability with an AUC-ROC of 0.64 for recovery prediction [8]. The recent study found unfavorable recovery with SPAN-100 in all age and older groups [29]. The recent study found that an NIHSS of 12 was a cutoff point for predicting functional outcome after thrombolysis [30,31]. 

Our study explored different scores with different probabilities of functional recovery. A previous study in Japan found that a younger age, lower initial NIHSS score, absence of internal cranial artery occlusion, higher ASPECTS on CT, and absence of intravenous antihypertensive agents before intravenous thrombolysis were associated with good recovery at 3 months with low-dose intravenous thrombolysis treatment [32]. Our study demonstrated that age and the NIHSS score within 3 h of stroke onset could predict functional recovery after intravenous thrombolysis in AIS patients with good performance. 

The crude OR of atrial fibrillation was 0.5079 (95% C.I.: 0.2311 to 1.1164), which seems to explore the trend of poor outcome with intravenous thrombolysis, but showed an insignificant difference. In addition, multivariable logistic regression with forward selection excluded atrial fibrillation. The logistic regression with added atrial fibrillation was no better than age and NIHSS (*p* = 0.5). Previous studies have found that atrial fibrillation is related to age in patients with sepsis and chronic obstructive pulmonary disease [33,34]. The older AIS patients (more than 65 years old) had more atrial fibrillation in our training dataset (*p* ≤ 0.001), and older patients with AIS had more atrial fibrillation reducing the influence of unfavorable functional recovery. The preventive treatment of embolic stroke with atrial fibrillation with anticoagulation therapy has educated physicians on stroke treatment and reduced recurrent stroke in recent years. The type of lacunar infarction in patients with AIS has better recovery [35]. 

**Figure 5:**
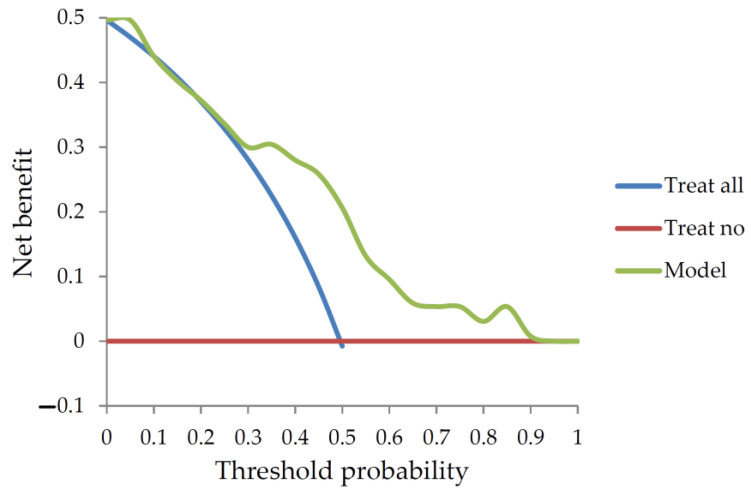
Decision curve analysis of the training dataset.

We selected an mRS ≤ 2 after 3 months of follow-up after treatment as a cutoff point; the ability of a patient to walk without support is indicative of a good quality of life. The median NIHSS was 15 among patients in the training dataset with a higher stroke severity, but these data points allowed for a better understanding of the impact of the stroke severity on the outcome. External validation was performed using a new independent dataset from a different institution, which allowed valuation of the utility and the applicability of the developed model [17]. The clinical net benefit was 0.21 in the threshold probability of 0.5 using decision curve analysis as shown in Figure 5.

Stroke is a complex entity. The effectiveness of intravenous thrombolysis treatment for AIS has been established. However, the ability to predict the outcome before treatment is limited. Although the benefits of intravenous thrombolysis treatment are difficult to assess at an early stage, we designed a user-friendly nomogram for outcome assessment with good performance to support outcome prediction. A prognostic model needs to be validated and easy to use for clinical application. Therefore, a multifactorial approach may be the best option to identify the response to intravenous thrombolysis. We developed a prediction model with adequate discrimination power and good calibration. We also validated the model on a community dataset with a performance that was better than the AUC-ROC of 0.775 achieved using only the baseline NIHSS in a previous study [31] and was similar to the complex model proposed by the Third International Stroke Trial that used age and the NIHSS score to predict unfavorable functional recovery with an AUC-ROC of 0.80 [36]. We used a cutoff point of 12 in the NIHSS score, and the SPAN-100 (Age plus NIHSS equal 100) found a lower AUC-ROC in the training and validation datasets. A summary of our study compared with the related studies is shown in Table 4.

Using this nomogram as semantic visualization to analyze individual outcomes based on age and the NIHSS score can offer a functional recovery probability prediction instead of only a favorable or unfavorable classification. According to this integer-based visual nomogram, stroke treatment physicians can better identify potentially good recovery benefits for patients and discuss these effective treatment options. A previous study in Japan found that good recovery was associated with no hypertension history or recanalization [37]. For patients who are predicted to have unfavorable outcomes by this nomogram as semantic visualization, the possible reason could be severer occlusion with ineffective recanalization, and aggressive management to achieve recanalization could be arranged for these patients. 

This study has some limitations. First, the stroke volume affects the stroke outcome, but brain magnetic resonance imaging is not necessary for intravenous thrombolysis in the emergency department without routine stroke volume data. Second, because 131 patients were included in the training dataset and 54 patients were included in the validation dataset, a larger cohort of patients is needed for a future verification analysis. Third, all patients received thrombolysis therapy under the guidelines of the Taiwan Stroke Association. However, low-severity stroke patients were enrolled in the validation dataset because we were limited to only two on-duty neurologists involved in stroke treatment consultations with selection bias that may also have led to a higher AUC-ROC. Patients with more severe AIS were sent to a higher level of medical instruction for treatment by emergency medical services in Taiwan. Intravenous thrombolysis data from the general population are needed to validate this finding. Fourth, the lower severity of AIS in the validation dataset showed better predictive performance. The aim of validation was to check the accuracy of the prediction model, which was 87% correct in unfavorable cases (28% of the total) that may have been overestimated. A prospective study must include more severe AIS patients to validate this nomogram in the future. In addition, although pulmonary embolism or mechanical ventilation used after intravenous thrombolysis may affect recovery, our study focused on recovery prediction from the factors of patients in the emergency department.

## 5. Conclusions

This study used a nomogram as semantic visualization for functional recovery prediction and included external validation in a community hospital. This study provided an applicable prognostic model with good performance for the prediction of the 3-month functional recovery after intravenous thrombolysis for individual patients in Taiwan based on age and the NIHSS score within 3 h of stroke onset but prior to treatment. Risks and benefits should always be balanced before any treatment is applied. Individualized recovery probability prediction can help stroke treatment physicians inform and discuss treatment options with patients and their families to reduce their anxiety about the outcome of treatment. During the hyperacute phase of ischemic stroke, intravenous thrombolysis may be recommended if the patient is predicted to have a favorable response, whereas a mechanical thrombus removal in large vessel occlusion may be considered for patients who are predicted to have poor recovery based on the nomogram as semantic visualization.

## 6. Patents

This section is not mandatory but may be added if there are patents resulting from the work reported in this manuscript.

## Figures and Tables

**Table 1:** Different characteristics between the two groups in the training dataset.

	Unfavorable (66)	Favorable (65)	*p*
Age	67.29 ± 9.98	59.94 ± 12.99	0.0004 *
NIHSS score	18(7–25)	13(7–27)	<0.0001 *
Hemoglobin	13.98 ± 1.70	14.21 ± 1.65	0.432
Onset of treatment time	136 ± 34	123 ± 38	0.048 *
Dose (mg/kgw)	0.68 ± 0.1	0.67 ± 0.12	0.539
Estimated GFR	78.18 ± 27.75	88.53 ± 31.18	0.047 *
Gender (Male)	38 (57.6%)	40 (61.5%)	0.776
ICA occlusion	10 (15.2%)	7 (10.8%)	0.627
Hypertension	51 (77.3%)	39 (60%)	0.052
Diabetes mellitus	17 (25.8%)	9 (13.8%)	0.136
Previous stroke	8 (12.1%)	12 (18.5%)	0.444
Hyperlipidemia	44 (66.7%)	49 (75.4%)	0.365
Coronary artery disease	17 (25.8%)	13 (20%)	0.565
Atrial fibrillation	33 (50%)	18 (27.7%)	0.015 *
Anemia	7 (10.6%)	7 (10.8%)	0.8
Middle cerebral artery sign	21 (31.8%)	10 (15.4%)	0.0778
Hospitalization survey			
Stroke type			0.538
Embolic	34 (51.5%)	27( 41.5%)	
Large vessel	28 (42.4%)	25 (38.5%)	
Lacunar	4 (6.1%)	13 (20%)	
Intracranial hemorrhage	26 (39.4%)	3 (4.6%)	<0.001 *

NIHSS score: National Institutes of Health Stroke Scale score. GFR: glomerular filtration rate. ICA: internal carotid artery. * *p* < 0.05.

**Table 2:** Patient characteristics in the training and validation datasets.

	Training	Validation	*p*
Total	131	54	
Age	63.64 ± 12.1	63.74 ± 10.12	0.96
NIHSS score	15 (7–27)	12 (4–26)	0.0003 *
Gender (Male)	78 (59.54%)	26 (48.15%)	0.287
Recovery	65 (49.62%)	39 (72%)	0.008 *
Survival	116 (88.55%)	51 (94.44%)	0.3385

NIHSS score: National Institutes of Health Stroke Scale score. * *p* < 0.05.

**Table 3:** Logistic regression in the training dataset.

Factors	Odds Ratio (95% C.I.)	*p*	Adjusted Odds Ratio (95% C.I.)	*p*
Diabetes mellitus	0.342 (0.122 to 0.956)	0.033 *		
Age	0.95 (0.917 to 0.984)	0.002 *	0.9477 (0.9155 to 0.9809)	0.0023 *
NIHSS score	0.861 (0.792 to 0.936)	0.0002 *	0.8643 (0.7997 to 0.9342)	0.0002 *
Anemia	0.611 (0.4228 to 4.325)	0.61		
Hypertension	0.4889 (0.2074 to 1.1523)	0.0986		
Gender (Male)	0.8777 (0.4039 to 1.9072)	0.7417		
Previous stroke	1.8214 (0.5998 to 5.5314)	0.285		
Atrial fibrillation	0.5079 (0.2311 to 1.1164)	0.0887		
Middle cerebral artery sign	0.4181 (0.1741 to 1.0041)	0.046 *		
Stroke type				
Embolic	reference			
Lacunar	4.093 (1.197 to13.992)	0.025 *		
Large vessel	1.124 (0.537 to 2.354)	0.756		

NIHSS score: National Institutes of Health Stroke Scale score. C.I.: confidence interval. * *p* <0.05.

**Table 4:** The performance of this study and comparison with the related studies. The performance of Stroke Prognostication using Age and NIH Stroke Scale (SPAN)-100 index and the threshold NIHSS score of 12 used training and validation compared with the current nomogram.

	Parameters	Cases	Sensitivity and Specificity	Area under ROC (95% C.I.)	Probability of Good Outcome	Semantic Visualization
Current study	Age, NIHSS	Training dataset:131	72.31% and 69.1%	0.753 (0.671–0.825)	Nomogram	Yes
		Validation dataset:54	71.79% and 86.67%	0.867 (0.765–0.968)		
SPAN-100 used current datasets (compared with nomogram)	Age + NIHSS = 100			Training: 0.515 (0.416–0.614), *p* < 0.001; Validation: 0.533 (0.356–0.711), *p* < 0.001		
NIHSS used current datasets (compared with nomogram)	NIHSS = 12			Training: 0.659 (0.565–0.753), *p* = 0.01 Validation: 0.674 (0.515–0.843), *p* = 0.004		
SPAN-100 [8]	Age, NIHSS	644		0.64		No
recent SPAN-100 [29]		1002	27% and 96%	0.74 (0.71–0.77)		No
Major neurological improvement [19]	Female	219		0.77 (0.7–0.79)		No
	Lack of cortical involvement					
	Glu < 8 mmol/dL)					
DRAGON [7]	Age, Glu, OTT, Dense sign, NIHSS	1319		0.84 (0.8–0.87)		No
				Internal validation: 0.8 (0.74–0.86)		
iScore [24]	Age: 1 every one-year-old	1696			≦139: more than 50%	No
	Gender: male:10					
	NIHSS: 9–13:40; 14–22:65; >22:105					
	Subtype: non-lacunar:30; undeterminated:35					
	Af:10					
	CHF:10					
	Cancer:10					
	Dialysis:35					
	Dependent:15					
	Glu > 150 mg/dl:15					
ASPECTS [27]	Early change of computer tomography of brain:	156	78% and 96%			No
	Basal ganglion involvement:3					
	Middle cerebal artery involvement:7					
TURN [28]	−4.65 + (mRS × 0.27) + (NIHSS × 0.1)	303		0.8 (0.74–0.85)		No
NIHSS [31]		11632	69.4% and 73.4%	0.775		No

NIHSS score: National Institutes of Health Stroke Scale score. C.I.: confidence interval. mRS: modified Rankin Scale.

## Data Availability

The datasets used in the current study are available from the corresponding author upon reasonable request.
